# Current state of ‘lost in the mall’: implications for expert witness testimony on the reliability of childhood memories

**DOI:** 10.1080/13218719.2025.2556461

**Published:** 2025-10-13

**Authors:** Michelle Drouin, Jody M. Ross, Tara L. Cornelius

**Affiliations:** ^a^ Department of Psychology, Purdue University Fort Wayne, Fort Wayne, IN, USA; ^b^ Health Services and Informatics, Mirro Center for Research and Innovation, Fort Wayne, IN, USA; ^c^ Department of Psychology, Grand Valley State University, Allendale, MI, USA

**Keywords:** childhood memories, expert witness, false memories, lost in the mall, memory distortion, memory evidence, memory implantation, witness memory

## Abstract

The seminal *Lost in the Mall* study by Loftus and Pickrell (^1995^) marked a pivotal shift in psychological science, reshaping how memory, and particularly false memory, is understood and researched. Over the past 30 years, it has inspired numerous replications and conceptual extensions of its methodological paradigm. Recently, a registered replication of the original study was conducted and their data were made publicly available, igniting renewed scholarly interest and debate regarding the nature, prevalence and classification of false memories. This article reviews the historical context of this body of research, synthesises key empirical findings and highlights recent studies that have once again positioned *Lost in the Mall* at the centre of memory science discourse. Moreover, we explore the implications of this research for legal proceedings and outline how it could be utilised in expert testimony, including the nuanced parameters that should be considered when discussing the psychological science of false memory.

## Introduction

One of the most widely debated topics in psychological research history is centred on the implantation of false memories and the veracity of ‘recovered’ memories, a notion popularised by Loftus and Pickrell (^1995^) in their seminal *Lost in the Mall* study. In this experiment, Loftus and Pickrell presented 24 students at the University of Washington a false story that they had been lost in the mall as a child, and approximately 25% of them adopted this false memory. The *Lost in the Mall* study was quite influential in the zeitgeist at the time, demonstrating empirically that suggestion, innocuous social pressure and repeated cognitive rumination on events can induce false memories. Moreover, *Lost in the Mall* showed that it is possible to insert a false autobiographical memory, that the person will believe it, and, presumably, these ‘memories’ will appear authentic and convincing to others. These findings seem to be especially relevant in legal cases wherein adults recover memories of child abuse. According to Loftus and colleagues (Ceci & Loftus, ^1994^; Loftus & Ketcham, ^1994^), these false memories are potential byproducts of therapists engaging their clients in memory recovery work or suggestions of abuse in therapeutic settings, a notion supported by a recent review of abuse allegation retractors (Otgaar et al., ^2025^). Notably, the *Lost in the Mall* study is still one of the most highly cited papers in psychology (number of citations as of July 2025 = 2064, Google Scholar citations) and is regularly discussed in both research and practice settings. It also continues to be enormously influential in legal contexts; Elizabeth Loftus estimated that she, herself, had consulted or testified in approximately 300 civil and criminal cases involving memory, including high-profile cases such as the Menendez brothers case and those involving Martha Stewart and Michael Jackson (Garcia, ^2017^).

However, almost as soon as it was published, the *Lost in the Mall* study began to generate criticism from researchers around the world, especially those involved in the treatment of trauma and recovered memories (Blizard & Shaw, ^2019^; Crook & Dean, ^1999^; Sinason, ^2022^). Some critics claimed that the research was fraught with methodological limitations, including small sample size, lack of operational definitions of the coding scheme and full/partial memories, and the sole reliance on experimenter determinations of false memories. Crook and Dean (^1999^) even went so far as to call the study a ‘breach of professional ethics’, citing concerns about the research methodology and the failure on the part of the original authors to correct sensationalised media claims about the findings. This camp, which included individuals from diverse areas of practice and research, has been vocal in their concerns regarding the wide application of these findings in legal contexts. As an example, Pope (^1996^) noted an appellate decision (i.e. State v. Warnberg, ^1994^) wherein the appellee asserted that a complainant suffered from ‘false memory syndrome,’ and the appellee requested an expert witness who could testify to this phenomenon. Pope warned that any opinions offered on the matter of false memories should be grounded in scientific principles, and that these principles should continue to be examined and tested.

In this spirit, and likely due to the fervour it created, the *Lost in the Mall* paradigm has been tested in a number of different age groups and samples over the last three decades, with varying methodologies to address some of the main criticisms of the original study. Then, in 2023, in response to continued criticisms of the methods of the original research (e.g. small sample size, lack of operational definitions of the coding scheme of full/partial memories and sole reliance on experimenter determinations of false memories), Murphy and colleagues undertook a task informed by decades of discussion: a preregistered replication of the original study. Seemingly settling the longstanding debate, Murphy, Dawson, et al. (^2023^) found results similar to Loftus and Pickrell’s original (1995) study: 35% of their participants were judged to have adopted the false memory. Additionally, as advocated by the open science movement and supported by those in the field of psychology and the law (Fessinger et al., ^2025^; Wiechert et al., ^2024^), some researchers in the false-memory arena, including Murphy, Dawson, et al. (^2023^), have made available their data sets, coding schemes and other research materials for scrutiny and reanalysis by others. Recently, the Murphy, Dawson, et al. (^2023^) results were re-analysed by Andrews and Brewin (^2024^), which spurred a rebuttal from Murphy and Greene (^2025^) and members of Loftus’s camp (Wade et al., ^2025^), reigniting this long-standing debate.

As this work has critical relevance to court testimony regarding traumatic memory, recovered memory and memories from childhood generally, a review of the major milestones and current state of this research is necessary. Experts in the courtroom, particularly practitioners who do not regularly examine the psychological research literature, may not be up to date on the breadth of false-memory implantation research of the past few decades, the recent registered replication that has reinforced Loftus and Pickrell’s original study (i.e. Murphy, Dawson, et al., ^2023^), and researchers’ attempts to recategorise or reclassify false memories (i.e. Andrews & Brewin, ^2024^). To provide comprehensive and competent testimony, expert witnesses must have an understanding of the origin of this work within the context of memory research, the studies that have been published since Loftus and Pickrell’s (^1995^) seminal work and the current research that is again challenging our understanding of childhood memories. Thus, the aim of the current work is to provide an overview of this body of literature for application in the courtroom.

## The nature of false memories

One of the most influential researchers on the fallibility of human memory over the last 25 years is Daniel Schacter (Schacter, ^2001^, ^2013^, ^2022^). According to Schacter, the human mind is vulnerable to sins and illusions that omit or distort reality. Two of those sins – transience and blocking – are especially relevant to memories of childhood abuse, as these traumatic childhood memories might be forgotten with the passage of time (transience) or be unable to be accessed (blocking). This blocking might take place consciously or unconsciously, through the process of dissociation or repression (e.g. Brewin & Andrews, ^1998^), though most memory experts are sceptical that repression of traumatic memories often occurs, despite its wider endorsement by laypeople, clinical practitioners, and hypnotherapists (Otgaar et al., ^2019^; Patihis et al., ^2021^). True childhood sexual abuse (CSA) experiences also might be plagued by memory deficits particular to traumatic experiences, such as flashes of memory (as opposed to complete memories), dissociation and memories that are not anchored to specific times or spaces (Jacobs-Kayam & Lev-Wiesel, ^2019^). Indeed, research has suggested that traumatic events might be *more* susceptible to memory distortion because they are accessed more often, either explicitly or internally, which may increase the potential for mental imagery distortions and source monitoring errors (Strange & Takarangi, ^2015^). On the other hand, benign or positive memories of childhood might also be distorted to more sinister representations through three other sins of memory: suggestibility (the influence of false information), bias (experiences, feelings or cognitions affecting past memories) or misattribution (assigning the incorrect source to a memory; Schacter, ^2001^, ^2013^, ^2022^). This might be exacerbated by misinformation, which can both cloud existing memories and create memories where they previously did not exist (Loftus, ^2004^; Loftus & Hoffman, ^1989^), a susceptibility that appears to increase with the passage of time, as memories fade (Loftus et al., ^1978^).

These sins of memory do not apply only to memories from long past. Even memories of recent events are subject to reconstructive bias, in part, due to the neurochemical processes involved in memory formation and storage (Schacter, ^2012^). Moreover, every time a memory is accessed, it is subject to change, and this becomes a never-ending reconstructive process (McKenzie & Eichenbaum, ^2011^). Recently, neurochemistry research has added more fuel to this debate by demonstrating that previously stored memories can change at the neural level when new information is processed (Zadbood et al., ^2022^).

With regard to the implantation of a completely false memory, such as the case for the *Lost in the Mall* paradigm, Hyman and Kleinknecht (^1999^) drew on the source monitoring framework (Johnson et al., ^1993^) and proposed that three conditions must be present to adopt a false memory. Specifically, the subject must: (a) accept the plausibility of the event, (b) construct a memory of the event (e.g. as a narrative or imagery), and (c) commit a source monitoring error (i.e. incorrectly attribute their memory construction to personal experience rather than to what was suggested to them). Mazzoni et al. (^2001^) revised this model, proposing that after establishing plausibility, the participant must believe that the event likely happened and then adopt any images or thoughts about the event as autobiographical memories. This model was further refined in a sophisticated analysis by Scoboria et al. (^2004^) wherein the researchers statistically examined the nested constructs of plausibility (subdivided into general and personal plausibility), belief and memory and found that these are distinct but related constructs. According to these latter theoretical models, the provoked false memory is adopted by the subject, who then appears to believe the memory is true. True belief of a false memory, prompted by provocation or spontaneously generated, is referred to as confabulation (Huntley & Brown, ^2015^; Wiggins & Bunin, ^2023^), which is a common and critical issue for legal professionals within court contexts (Brown et al., ^2017^).

## Implanted false memories: beyond Lost in the Mall

In the original *Lost in the Mall* study in 1995, Loftus and Pickrell utilised a memory implantation paradigm with 24 college students recruited for a study on childhood memory. To create the materials for the study, a relative (parent or adult sibling) was recruited to provide real stories from the participant’s childhood that happened between the ages of 4 and 6. The participant was then mailed a booklet with four short childhood stories, three of which were true and one ‘Lost in the Mall’ story that was fabricated by the researchers to contain the same main points across participants (i.e. child was about 5 years old, became lost in a mall or department store, was lost for an extended time, was found crying by an elderly woman, and was reunited with family) but tailored to the individual person based on details provided by the relative (e.g. with whom and where the participant usually shopped). Loftus and Pickrell also confirmed with the relative that the participant was never lost in the mall at around age 5. Participants were then asked to read the four stories and write what they remembered about each or state that they did not remember. After they mailed the completed booklets back, participants were interviewed by phone or in person twice (1 to 2 weeks after the booklet was received and 1 to 2 weeks after the initial interview) and were asked to provide as much detail as they could about the events in the booklet, including details they may not have provided in their written narratives. During each of these interviews, participants were also asked about how clear their memory of the event was (1 = *not clear*, 10 = *extremely clear*) and how confident they were that if given more time they would remember more details (1 = *not confident*, 5 = *extremely confident*). At the end of the second interview, the participants were debriefed and were asked which event they thought was false. Since *Lost in the Mall,* there have been numerous studies that have attempted to recreate this procedure in varied settings and with alternative false memories (see Table 1 for those described below). Many of these improved upon aspects of the original study in order to address methodological concerns and increase real-life applicability.

**Table 1.** Selected implanted memory studies since Lost in the Mall.

Study	Sample	False-memory scenarios	True event recall	False event recall	Key findings
Hyman et al. (^1995^) – Study 1	20 undergraduates (15 females)	Clown at birthday,hospitalisation for ear infection	83.8% (1st interview), 91.9% (2nd interview)	0% (1st interview),20% (2nd interview)	Memories can be implanted for both negative and positive events.
Hyman et al. (^1995^) – Study 2	51 undergraduates	Spilling punch on bride’s parents, sprinkler activation, car rolling incident	88.7%, 93.2%, 95.1% (across 3 interviews)	0%, 17.6%, 25.4% (across 3 interviews)	Even for negative events unlikely to occur, false memories can be implanted.
Porter et al. (^1999^)	77 undergraduates	Serious animal attack, getting lost, indoor/outdoor accident	97.4%	26% (complete memory), 30% (partial memory) across 3 interviews	Serious, traumatic memories from childhood can be implanted.
Wade et al. (^2002^)	20 undergraduates (10 females)	Pictorial depiction of a hot air balloon ride	93.3% (1^st^ interview) 96.7% (3^rd^ interview)	35% at 1^st^ interview(1 full, 6 partial) 50% at 3^rd^ interview(4 clear; 6 partial)	False-memory implantation can occur with false photos, perhaps at a higher rate and with increased confabulation over time.
Johnson et al. (^2023^)	20 undergraduates (10 female)	Pictorial depiction of a Viking ship ride	87% (1^st^ interview) 95% (3^rd^ interview)	20% at 1^st^ interview(1 full, 3 partial) 40% at 3^rd^ interview(6 clear; 2 partial)	Replication of pictorial false memories, demonstrating increased elaboration of memories over time.
Shaw and Porter (^2015^)	60 undergraduates	Criminal behaviourbetween ages 11–14	Not reported	70% reported criminal behaviour	Suggests that utilising suggestive interview techniques, rich and detailed accounts of false criminal behaviour can be generated.
Wade et al. (^2018^)	Reanalysis of Shaw and Porter (^2015^)	Criminal behaviourbetween ages 11–14		26–30% reported false memories	Differentiating between false memories and false beliefs is critically important; false memories are consistent with previous research.

Close in time to the original study, Hyman et al. (^1995^) tested a variant of the Loftus and Pickrell (^1995^) paradigm, involving a childhood event at age 5 that was either positive (i.e. a clown visiting the child’s birthday pizza party) or negative (i.e. the child spending a night in the hospital due to an ear infection) with a similar protocol wherein the researchers also presented real events recalled by parents. Within their sample of 20 psychology undergraduates (75% female), participants recalled 83.8% of the true events at first interview and 91.9% of true events at second interview. In contrast, no one recalled the false events at first interview, and 20% incorporated descriptions of the false events in the second interview. In a second experiment with a different undergraduate sample (*N* = 51, 67% female), Hyman et al. tested three scenarios: spilling a punch bowl on a bride’s parents at a wedding, a fire sprinkler system activated while shopping with a parent, and the child (left in the car while parent was in a store) playing with the parking brake, resulting in the car rolling into something. The researchers also provided more cues and details of the false events and emphasised to the participants that they were expected to provide full details. Results were similar for memory of true events (88.7%, 93.2% and 95.1% across three interviews) and memory for false events (i.e. 0%, 17.6% and 25.4% across the three interviews). Hyman et al. suggested that the increase in false-memory recounts over time could be a result of a source monitoring error, wherein the participants attributed any memory of the event to childhood rather than the experimenter’s suggestion.

Soon after, Porter et al. (^1999^) utilised the implantation protocol with 77 undergraduates (*M_age_* =19.2 years, 79.2% female) using more emotionally negative events from parents, such as a serious animal attack, getting lost, or an indoor or outdoor accident. In this case, participants were presented with two childhood events, one real and one that was not experienced (as confirmed by parents) but contained details from the parents’ reports, such as ages, locations and other cues that would fit with a potential real memory. During the initial interview, participants received four details from the real or false event (counterbalanced by participant), and they were asked what they recalled about the event. If they failed to recall the real event, they were presented with a backup real event. If they failed to recall the false event, they were socially pressured to remember more, instructed on guided imagery, and encouraged to think more about the event each night. Overall, 97.4% of participants recalled the real event (either the first one presented or a backup real event). Meanwhile, across the three interviews, 26% generated a complete memory, and 30% generated a partial memory of a false event; of those who generated a false memory, 35% did so in the initial interview, 50% in second interview, and 15% in third interview. However, and importantly, these implanted false memories were judged by the participant as less vivid and stressful, and they had less confidence about the event and provided fewer details than for real memories.

While the previous research had demonstrated that detailed descriptions provided by caregivers could elicit false memories in many participants, other researchers have extended the false-memory research beyond personalised narratives to false photographic stimuli (Hessens-Kayfitz & Scoboria, ^2012^; Lindsay et al., ^2004^; Wade et al., ^2002^). In the first known study using this photographic paradigm, Wade and colleagues utilised image-manipulation technology to implant a false memory of a hot air balloon ride, utilising actual photos of the participants as children and electronically importing this into the false image. Consistent with other research, the undergraduate participants were also shown three digitised photographs of true, positively valenced events from their early childhood, along with the doctored new event. Participants (*N* = 20; *M_age_* = 20.2, 50% female) were interviewed three times over a period of approximately 2.5 weeks and were asked to report everything they could recall about the pictorial events. Across the three interviews, participants recalled more than 90% of the true memories, and by the conclusion of the third interview, 50% of participants were classified as adopting the false memories (4 ‘clear’, 6 ‘partial’ memories). Additionally, analysis of participants’ false-memory descriptions indicated that memories for the false event evolved over time, with initial fragmented reports and then subsequent embellishment over time. In fact, by the final interview, participants were reporting details about the setting, their perceptions and emotional responses that were not represented in the photographic depictions. This research was recently replicated in Norway by Johnson et al. (^2023^), utilising a false pictorial representation of a culturally relevant Viking ship ride, and results were largely consistent: participants (*N* = 20; *M_age_* = 21.7, 50% female) recalled approximately 90% of true events across the interviews, and by the third interview, 40% were classified as adopting the false event (6 ‘clear’, 2 ‘partial’ memories). Similarly, participants developed rich additional ‘memories’ beyond the false photograph, to include mental imagery, emotional responses and coherent elaborations of the alleged event, demonstrating the constructive nature of memory, even for false pictorial events.

Finally, Shaw and Porter (^2015^) garnered attention with their research demonstrating implantation of full false episodic memories of adolescent criminal behaviour in 70% of their undergraduate participants, a rate much higher than previous research. Utilising the basic memory implantation paradigm, participants (*N* = 60; *M_age_* = 20), completed three interviews spaced approximately 1 week apart in which they were presented with two events, one true and one false, that occurred between the ages of 11 and 14, as verified by a parental informant. Participants were randomly assigned to either a false event of a criminal nature that resulted in contact with the police (i.e. committing an assault, an assault with a weapon or a theft) or a false event about a non-criminal, emotionally valenced event involving self-injury, a dog attack or the loss of a substantial amount of money followed by getting into trouble with their parents. Interviewers used methods that have been demonstrated to elicit false confessions, including social pressure, long pauses, presumed additional knowledge, providing participants with incontrovertible evidence, and suggestive retrieval techniques, such as mental imagery. Of the participants assigned to the criminal condition, by the third interview 70% reported ‘rich’ false memories of criminal behaviour, to include assault, assault with a weapon and theft. Consistent with other research (Wade et al., ^2002^), the participants generated novel details of the event, to include descriptions of law enforcement personnel and their cognitive/emotional responses to the false event. As such, this research generated extensive confabulation of richly detailed recollections of false criminal behaviour. However, these findings have been criticised by other researchers, as the reported rate of false memories represents a notable outlier compared to the average of 22% identified in the Scoboria et al. (^2017^) mega-analysis. Further, Wade et al. (^2018^) note that this research fails to differentiate between false memories (when a person confidently claims to remember the event, potentially including elaborations or emotions) and false beliefs (when a person accepts that the event happened or can imagine that it did), which creates fundamental limitations in interpreting the findings. In their reanalysis of the Shaw and Porter data utilising established coding schemes, the rates of false memories were identified by Wade and colleagues to be much lower: 30% reporting false memories of criminal events under one coding scheme (Lindsay et al., ^2004^) and 26% under another (Scoboria et al., ^2017^). Additionally, in several subsequent analyses of the Shaw and Porter data with laypersons (versus trained coders), the data converged on the rate produced by the Lindsay et al. (^2004^) coding scheme, suggesting that approximately 30% of untrained participants judged the person as actually remembering the suggested event. Wade et al.’s (^2018^) data are more in line with previous research and demonstrate that a significant proportion of the participants in Shaw and Porter may have simply accepted, speculated upon or conjured mental imagery of the suggested event, rather than developing a false recollection.

Although a comprehensive review of every published article in the false-memory implantation literature is beyond the scope of this review, we have summarised a subset of studies with adult participants to provide the reader with a basic understanding of the methodology and pattern of findings in this body of research. The findings summarised here are in line with other published outcomes related to the feasibility of false-memory creation using widely employed paradigms. Other research that includes the use of self-relevant details or suggestive interviewing to enhance false-memory generation (e.g. Desjardins & Scoboria, ^2007^; Oeberst et al., ^2021^) may provide additional context for the courtroom, and the reader is directed to these additional references for further review.

## The replication study and its reanalysis

Clearly, the foregoing research has generated much interest in false-memory formation. Though much research on the implantation of false memories has been conducted over the last 30 years, recently, Murphy, Dawson, et al. (^2023^) conducted a full, preregistered replication and extension of the original *Lost in the Mall* study. This research addressed methodological limitations that are particularly relevant to the legal setting, namely the participants’ perceptions of memory and veracity of the false memories, as well as the presence of sensory details with regard to the false memories. Because Loftus and Pickrell’s (^1995^) original research relied solely on external evaluations as to the degree to which an individual remembered the event, this extension was deliberately designed to test whether the participants themselves also articulated the veracity of those memories. This is critically important in the context of legal or administrative proceedings, as these hearings may be predicated on an individual actually believing or coming to believe the events happened and then reporting on these to the factfinders in the case. Additionally, they extended the research by examining the degree to which the false memories were judged to be authentic and believable in a separate sample of mock jurors. The legal implications of this, coupled with research that demonstrates that false memories are largely indistinguishable from authentic ones (Shaw, ^2020^), makes this central to the ecological implications of these findings.

The procedures of the Murphy, Dawson, et al.’s (2023) replication were largely identical to those in the original Loftus and Pickrell (^1995^) research, but Murphy et al. recruited a larger sample (*N* = 123; M_age_ = 25.49; 66% female), deliberately excluded psychology students, and altered the procedures to utilise modern technology (computerised ‘booklets’ and video interviews). The role of the informant-relative was identical, including confirming that the participant had not been lost in the mall during childhood, and the order and timing of the stimuli, booklet and interviews were identical. Participants rated, in addition to clarity and confidence, whether the memory elicited sounds, emotions or visual imagery, and the ease at which they could access the memory. They also were asked the degree to which they remembered the event, and whether they believed the event really occurred. Murphy, Dawson, et al. (^2023^) also significantly improved on the coding scheme for classification of the memory as ‘full’ or ‘partial’ (see Table 2), by providing operationalisation, extensively training coders, and ensuring interrater reliability (κ = .60), which was not included in the Loftus and Pickrell (^1995^) study. Finally, to verify the ethical treatment of participants, follow-ups showed that the debriefing was effective for the vast majority of participants (Greene et al., ^2024^), and both participants and informants were generally positive about their experiences and found the study ethical (Murphy, Maher, et al., ^2023^). These findings should assuage critics’ concerns about ethical barriers to this line of research.

**Table 2.** Detailed coding scheme from Murphy, Dawson, et al. (^2023^).

Memory type	Definition
Full memory	Participant substantially remembers the event, and may speculate about some peripheral details. For this category, it should be clear that the participant actually remembers the event, even if they can’t remember some minor details.
Partial memory	Participant remembers parts of the event (e.g. recalls some features of the event or peripheral details); participant may also speculate about how the event might have happened. *Note: some element of memory (however minor) is required for this code. Speculation alone is not enough if there is no indication of any memory.*
No memory	Participant clearly does not remember the event. They may speculate about some details in response to questions (e.g. ‘yeah, we used to go to that shop a lot’ or ‘I suppose my mam would have been upset if I was lost’) but do not remember any details of the event.

*Note:* From preregistration site: https://osf.io/b3xcw

In terms of the overall percentage of true and false memories reported, the results were largely consistent with the original Loftus and Pickrell (^1995^) research (see Table 3). Overall, 35% of participants were classified as having a false memory, although most of those formed partial memories, rather than full memories (27% partial, 8% full), which is largely consistent with the overall prevalence of 25% identified in the seminal research. However, while the original research suggested consistency over time, Murphy, Dawson, et al. (^2023^) found an increase in false memories over the repeated reporting events (booklet, Interviews 1 and 2). This is consistent with other implanted memory studies (e.g. Hyman et al., ^1995^; Porter et al., ^1999^) and aligns with the notion that increased questioning, imagining and thinking about the events may serve as reminders that alter the construction of stored memories (Hupbach et al., ^2007^), changing representations at the neural level (Zadbood et al., ^2022^). Additionally, and consistent with Loftus and Pickrell (^1995^), participants reported lower clarity and confidence in the false memories, which were less detailed, utilised fewer words and contained less sensory information than true memories (see also Porter et al., ^1999^).

**Table 3.** Alignment of results from Murphy, Dawson, et al.’s (2023) replication and Loftus and Pickrell’s (^1995^) study.

Memory type	Loftus and Pickrell (^1995^) Coded false (Interview 2)	Murphy, Dawson, et al. (^2023^) Coded false (Interview 2)			Murphy, Dawson, et al. (^2023^) Participant rated
	%	%	Partial	Full	%
True	68	87	98	223	75
False	25	35	33	10	14^a^

^a^An additional 52% believed it occurred, even if they could not remember.

Importantly, when Murphy, Dawson, et al. (^2023^) examined rates of false memory identified by the experimental coders compared to participant ratings, notable trends emerged. First, memories categorised by coders as full false memories appeared to be more convincing to participants themselves, as reflected by a greater likelihood that the individual reportedly ‘remembered’ the event (Murphy, Dawson, et al., ^2023^). Second, while coders rated 35% of the false event narratives to reflect memories (either full or partial), participants themselves reported a memory far less often (14%), although 52% reported that they did *believe* they had been lost in a mall, even if they did not have a memory of it specifically. In a legal context this might be particularly relevant in that individuals may not be entirely convinced of their false memory if, for example, it lacks perceptual richness, clarity or emotion as compared to other events in their memory; however, an independent observer might still conclude that the witness appears to be remembering a genuine experience.

The expansion data with the mock jurors is particularly significant here, as it assessed how these false memories are perceived by outside observers (not trained coders) who are faced with a description of the false memory. This research (*N* = 1006) involved exposing mock jurors – naïve participants – to false-memory transcripts and then assessing whether the mock juror (a) believed the event happened, (b) thought the person was describing an event they remembered, and (c) thought the person believed the event had happened. For the 111 false event extracts, they were rated as memories 39% of the time. Mock jurors also reported that they perceived the person to be remembering or believing the events 41% of the time, suggesting that reports of false events are perceived as credible and believable by others, which has important implications for factfinders in legal contexts. More than a third of the mock jurors rated the false memory as reflective of an actual memory, which was more than the participants themselves and those coded by the researchers. This suggests that false memories may be convincing to outside individuals, despite participants themselves being less sure of their veracity. Considered in its entirety, Murphy, Dawson, et al. (^2023^) largely replicated the original research and provide additional evidence that false memories can be implanted in participants and that outside observers may also believe that fully fabricated events did, in fact, occur.

Though the results of this research largely replicated the original Loftus and Pickrell (^1995^) overall findings, the discrepancy between the coders’ ratings of false memories and participants’ self-reported memory highlighted the need to consider the strengths and weaknesses of different coding methods. Thus, Andrews and Brewin (^2024^) conducted a granular analysis of Murphy, Dawson, et al.’s (2023) transcribed data to determine the factors that contributed to investigator-rated ‘partial’ and ‘full’ memories in the replication research. Unlike Loftus and Pickrell’s findings, Murphy, Dawson, et al.’s (2023) replication did include a coding scheme for full, partial and no memory, although it is unclear, operationally, what recollections would indicate ‘substantial recollection of the event’, for example. Andrews and Brewin aimed to examine whether those memories identified as false by the coders evidenced the ‘core details’ of the false event, to include being lost, as well as whether some participants’ narratives were reflective of potentially true experiences, based on the details they reported. Utilising the SPSS file and transcripts from Murphy, Dawson, et al. (^2023^), this research focused specifically on the narratives of the adopted false memories (*n* = 43) for the ‘core details’ of the implantation experimental paradigm. Those core details were sixfold and paralleled those from the original research (Loftus & Pickrell, ^1995^): (a) being lost for an extended time; (b) crying; (c) being lost in a large mall or store; (d) being found and assisted by an elderly female; (e) reunification with the family members; (f) age when lost. For each of the core details, recall was coded based on the extent to which the memory was recalled by the participant: Explicit Recall; Different Recall; Uncertain Recall; No Recall; No Mention for each of the core details. This was conducted in order to explore the actual content of the memories reported and the similarity of that content to the six identified core details. The results suggested that for the 43 individuals who were coded as having a false memory, the average number of core details that were explicitly, differentially or uncertainly recalled was 1.47. For those who were classified by Murphy, Dawson, et al. (^2023^) as having *full* false memories, the average number of explicit core details recalled was 2.70, with no participant reporting more than four of the core details. Additionally, even among those who were classified as having full false memories, 20% did not explicitly recall being lost as one of the core details. For those identified as having *partial* false memories, the average number of core details was 1.09, with approximately 39% not explicitly recalling *any* of the core details.

As noted in much of the research in this field, and as highlighted by Andrews and Brewin (^2024^), a significant limitation of this research is that participants could be recalling a true instance of being lost in a mall, perhaps one their caretaker was not aware of or had forgotten. Indeed, in Murphy, Dawson, et al. (^2023^), three participants, even after being debriefed, persisted in their belief that they had been lost, even after being confronted with the false event. It is possible that these false memories are an artefact of other similar, actual experiences that share similarities and differences to the implantation paradigm stimuli. Andrews and Brewin, through their reanalysis of these data, examined the transcripts to determine whether the details provided about the false events substantially reflected the implantation event, or whether the detail departed significantly, thereby suggesting a potential true event, rather than elaboration on the false one. Of the full sample, 30.9% described past experiences that were relevant, yet distinct, from the false event, to include being lost in a different location or situation, being abandoned, or being lost more than once. Further, those reporting false memories were significantly more likely to report similar prior life events than those who did not report a false memory, with 50% of those classified as having *full* false memories describing such potentially true prior life events, as did 51.5% of those judged to have *partial* false memories. A fundamental premise of the implantation procedure is that individuals who have experienced the event cannot be included in the research, since they may then be reporting on true events. The data examined here suggest that approximately half of the coded false memories evidenced distinguishable details from the false event, which could be indicative of reporting on a true event that had similar and distinct details. In fact, Andrews and Brewin ultimately conclude that, reporting in aggregate, experimenter-coded false memories may be confounded with individuals reporting true events and those that do not recall even being lost, and the rates of false memories from Murphy, Dawson, et al. (^2023^) may be substantially lower than their reported 35%, and perhaps as low as 4%. This reanalysis suggests that the premise that people can be influenced to remember entire events that did not happen to them may be overstated, as many of these ‘memories’ do not contain the majority of the core details, or even the core detail of being lost.

Recently, both Loftus and colleagues (Wade et al., ^2025^) and Murphy and Greene (^2025^) wrote commentaries responding to the Murphy, Dawson, et al. (^2023^) replication and Andrews and Brewin (^2024^) reanalysis. Wade et al. (^2025^) noted that the Murphy, Dawson, et al. (^2023^) replication and extension further validated the original false-memory results and demonstrated that these false memories can appear convincing to outside observers. Additionally, while recognising the inherent value in the reanalysis conducted by Andrews and Brewin, they expressed concerns over the conclusions that, if a memory contained remnants of a true experience, it was *ipso facto* indicative of a true, rather than false, memory. In fact, research has demonstrated that participants’ false memories are often littered with schematic knowledge or other autobiographical events (Johnson et al., ^2023^; Wade et al., ^2002^), to include details that are not included in the implantation stimuli. These findings reflect patterns of real childhood memories as well, with about 80% of details in true autobiographical memories differing from photographic evidence (Wade et al., ^2002^). Additionally, the large body of empirical research on the constructive nature of memory would suggest that most memories, even authentic ones, are likely to include inconsistent or erroneous details, often becoming distorted, exaggerated and changing over time (Goldfarb et al., ^2019^; Pezdek, ^2003^; Schacter, ^2013^; Tekcan et al., ^2003^). These researchers surmise that false memories would be subject to the same processes as genuine memories, with intrusions, omissions and inconsistencies, and this does not necessarily indicate either that the implanted false memory is really a true one, or that because a core detail is missing from their report, then it is not a false memory. Additionally, Wade et al. (^2025^) note that a large body of research using the implantation paradigm, over the last 30 years, has consistently demonstrated that even impossible or implausible event memories can be created, which should not be minimised based on one replication study reanalysis. Fundamentally, these authors note that while there is disagreement about the *rate* of false memories, the fact that they can be implanted is of central importance, and that is not a fact that is sufficiently challenged by Andrews and Brewin’s (^2024^) reanalysis.

Murphy and Greene (^2025^) echo these sentiments and further challenge the coding scheme used by Andrews and Brewin (^2024^). Though Murphy and Greene appreciated the further analysis of the 2023 replication study, which was one of their motivations for providing open access to their data and coding, they also emphasise that rates of false memory may differ substantially according to the coding scheme applied. With regard to the specific coding employed by Andrews and Brewin, Murphy and Greene challenge their count-based method of classification (see also Wade et al., ^2018^), in which they selected six core details for false-memory validation. This included the age of the participant (5 years old), which, according to Murphy and Greene, 0% of participants with full memories mentioned in their narratives. Hence, according to Murphy and Greene, some of the core details Andrews and Brewin selected for classification were not well chosen, arbitrary, and not likely to be produced within the natural flow of the participant’s conversational narrative. More importantly, the count-based classification scheme they utilised failed to capture the richness of detail in participants’ narratives, assigned equal weighting to each detail (e.g. both name of shopping mall and the fact that child was lost are given equal weight), and did not take into account the participants’ own assessments of memory clarity and belief in the veracity of the event. This latter point might be particularly important because, as mentioned previously, belief in the event is one part of the process in the development of false memories (Mazzoni et al., ^2001^; Scoboria et al., ^2004^)

## Full versus partial false memories: does this distinction matter?

As detailed, a key criticism of the original *Lost in the Mall* study was the lack of clarity in assigning the labels of ‘full’ or ‘partial’ false memories to participants’ event descriptions, a distinction Andrews and Brewin (^2024^) focused on in their reanalysis. Partial and full false memories, while both involving some acceptance of a fictional event, have been distinguished by factors such as degree of certainty and level of elaboration associated with an individual’s false event narrative.

As shown in Murphy, Dawson, et al. (^2023^), memories categorised as full false memories may seem more convincing to participants themselves, as reflected by a greater likelihood that the individual reportedly ‘remembers’ the event. This aligns with previous research showing that confidence ratings for full false memories are markedly higher than for partial false memories (Lindsay et al., ^2004^). In contrast, participants generating partial false memories tend to provide less detailed narratives and be less certain (Andrews & Brewin, ^2024^) or confident (Wade et al., ^2002^) that the event actually occurred. When third parties are provided summaries of full false memories, they may be more convinced as to their plausibility, though, again, a majority of ‘mock jurors’ have been shown to believe that an event happened whether the false memory had been coded as full or partial by researchers (Murphy, Dawson, et al., ^2023^). In sum, false-memory narratives, both partial and full, may be compelling to third parties, who may be more liberal in evaluating the authenticity of these narratives than are the individuals who generate them.

Even when definitions and processes have been made clearer, interrater reliability scores reflect the difficulty that sometimes arises in distinguishing between partial and full false memories (Murphy, Dawson, et al., ^2023^), though researchers expanding beyond this two-category scheme have reported higher agreement between coders (e.g. Johnson et al., ^2023^). Additionally, partial and full memories have been defined in various ways (see Scoboria et al., ^2017^) and are just two among several labels used to describe false memories. Terms such as ‘complete’, ‘robust’ and ‘accepted’ have also been used as false-memory labels (Calado et al., ^2021^; Scoboria et al., ^2017^; Shaw & Porter, ^2015^). In some research, memories of a false event are contrasted with images generated by participants related to the false event (e.g. Garry & Wade, ^2005^; Strange et al., ^2008^), which have been interpreted by some as comparable to ‘partial’ false memories (Desjardins & Scoboria, ^2007^; Lindsay et al., ^2004^) and by others as a category distinct from partial false memories (Hyman & Pentland, ^1996^). Furthermore, even when partial and full false memories are assessed, researchers have often summarised across these two categories in describing their results, despite warnings against this practice (Lindsay et al., ^2004^). If the partial versus full false-memory distinction is considered important, researchers should be encouraged to (a) identify an agreed-upon definition and coding scheme for these two categories, and (b) make explicit which of their findings apply to partial false memories, full false memories, or both.

Another methodological issue in the research examining the partial versus full false-memory distinction is the use of similar criteria to create and validate these categories. For example, in their mega analysis, Scoboria and colleagues (^2017^) differentiated full and partial false memories based on degree of elaboration and generation of imagery related to the suggested (false) event. Reflecting on this distinction in their results, the authors suggest that their ‘criteria appropriately and conservatively allocated participants with vague or fragmented memory representations into the partial memory group’ (Scoboria et al., ^2017^, p. 155). More recently, Murphy, Dawson, et al.’s (^2023^) distinction included that, for full false memories, the ‘participant substantially remembers the event’, while in partial false memories the ‘participant remembers parts of the event’. Support for this distinction included mock juror perceptions that full memories were relatively richer, including that they were clearer and more detailed than partial memories (Murphy, Dawson, et al., ^2023^). As described above, Andrews and Brewin (^2024^) identified six core details of the ‘fake story’ and reported that partial false-memory narratives included significantly fewer of these core details than the narratives categorised as full false memories. Partial false memories, then, are initially defined as being less substantial, elaborate and visually rich, and are then validated as distinct from full false memories by their relative vagueness, clarity and lack of detail. Perhaps the most important conclusion that can be drawn from the research into partial and full false memories is that they do exist; some individuals do generate false memories, which vary in terms of their level of detail, elaboration and imagery.

The partial/full distinction can be difficult to judge reliably (Murphy, Dawson, et al., ^2023^), and the definitions of and support for this distinction can be tautological, as described above. There is even inconsistency regarding which type of false memory is more common, with some studies reporting proportionally more partial false memories (Murphy, Dawson, et al., ^2023^; Porter et al., ^2000^; Wade et al., ^2002^) and others reporting higher rates of full false memories (Johnson et al., ^2023^; Scoboria et al., ^2017^; Shaw & Porter, ^2015^). Considering these issues, an alternative, dimensional approach reflecting the strength or ‘robustness’ of a false memory may be more useful in research than the current partial–full false-memory dichotomy. However, all of this research needs to be considered within the wider landscape of its application. If researchers, who are supposed to apply an objective, scientific lens to their judgments and classifications, are having difficulties with the categorisation of memories as partially or fully capturing the essence of the memory, how can layperson jurors make such determinations? And, more importantly, how can they take the additional step of determining its veracity? This makes especially salient the role of the expert witness, who must present an overview of the current state of the research, with the added caveat that whether a memory is full or partial does not give any indication of whether the event actually happened. As was posited by Thomson (^1995^, p. 103) and is still true today, ‘there is no clear way of discriminating between authentic memories and fabricated memories’.

## Discussion

The science of implanted memories is extremely important to the law. Within a court setting, jurors may have misconceptions about the nature of memory and be swayed by emotions or the conviction with which an individual recounts an incident, despite the fact that conviction and emotional response are not good indicators of the veracity of a memory (McNally, ^2003^; McNally et al., ^2004^). As such, experts in psychological science are often relied upon to clarify the nature of memory, memory distortion, confabulation and false memories (Otgaar et al., ^2022^). The previous discussion outlines the large body of research examining not only the phenomena of memory reconstruction and false memories, but also contextual factors that can serve as explanatory mechanisms for their manifestation.

If the decades of research in this field have taught us anything, it is that complexity and nuance in the research must be considered in expert testimony on the reconstructive nature of memory and false memories. Considering the amount of criticism the original *Lost in the Mall* study garnered (Blizard & Shaw, ^2019^; Crook & Dean, ^1999^; Sinason, ^2022^), it is wholly insufficient for experts to report only on the original research findings without exploration and analysis of more recent data in the field of psychological science on memory. Additionally, expert witnesses should be aware of and ready to face cross-examination on criticisms of methodology, to include those in the original *Lost in the Mall* study through the most recent replication by Murphy, Dawson, et al. (^2023^) and those studies that reanalyse its data. They may also be cross-examined on individual factors that could impact the degree of memory distortion, such as suggestibility, acquiescence and fantasy proneness (Eisen et al., ^2001^; Gudjonsson & Clare, ^1995^; Somer & Otgaar, ^2025^) and the application of these experimental findings to real-world contexts (Szojka et al., ^2025^). Educating attorneys about these potential cross-examination points and any relevant direct and redirect is critical to effective testimony.

Recent improvements in research, to include advanced statistical methods and the open science movement, enable expert witnesses to better present empirical data in aggregate and acknowledge the limitations of individual studies. Expert witnesses should be skilled at synthesis of the research and nuanced discussion of mega-analyses that have been conducted, and be prepared to explain, especially on cross-examination, how outlier data (e.g. Andrews & Brewin, ^2024^; Shaw & Porter, ^2015^) should be framed within the larger context of the body of empirical research (Otgaar et al., ^2022^). While Shaw and Porter’s (^2015^) findings certainly represent a statistical anomaly, a key takeaway from this research should be the impact of suggestive interviewing on false-memory elaboration and false confessions. Indeed, this research demonstrated that, in the face of more coercive interview methods, individuals may be especially likely to generate complex and multisensory reports of events that did not happen. Psychological science has long demonstrated the powerful impact of situational variables on behaviour (Mischel, ^1968^; Mischel & Peake, ^1982^), and a large body of research examines how these methods may elicit false confessions (Kassin et al., ^2012^, ^2010^). Plausible confabulations of criminal behaviour may be constructed from fragments of autobiographical memories, and these can include rather convincing elaborations that are either spontaneous or provoked by investigator questions. However, recognition of the nuances of human memory, situational pressure and prevailing models of memory formation and recollection should give pause to wholesale adoption of these memories as ‘truth’. As such, expert witnesses should be cognisant of the impact of interview techniques, methods, social tactics and coercive control that can elicit reports of false or distorted memories, and they should frame these phenomena within the existing research on the reconstructive nature of memory.

The generalisability of these laboratory experiments to specific autobiographical events is inherently limited (Szojka et al., ^2025^), and absent a recording of the event, it is often impossible to determine the accuracy of a memory in real-world contexts, such as legal proceedings. However, debates about the exact prevalence or likelihood of false memories may be tangential to the core application of these data. Murphy and Greene (^2025^) underscore this, suggesting ‘there is no absolute false memory rate’ (p. 2), but even a 4% false-memory rate has important theoretical and legal significance. Murphy and Greene further contend that actual rates of false memory may be even higher than the approximate 25% rate demonstrated in single studies, as autobiographical memory accuracy varies not only across individuals but also within individuals across different tasks (Murphy, Dawson, et al., ^2023^); hence, there does not appear to be a fixed ‘false-memory trait’, and all individuals are potentially susceptible to memory distortion (Patihis, ^2018^). Perhaps even more relevant to the courtroom, Murphy and Greene argue that laboratory studies typically utilise ‘light-touch’ memory implantation paradigms (brief discussions of an event over two occasions), which pales in comparison to the time counsellors, hypnotherapists, or even friends and family may discuss a fabricated event with someone who goes on to develop a false memory (see also Scoboria & Mazzoni, ^2017^; Wade et al., ^2025^). Therefore, witnesses who have had cause to recount suggested incidents prior to their in-court testimony (e.g. in therapy or through previous interviews with counsel or investigators) may be at an increased risk of generating false memories (Hyman et al., ^1995^); these kinds of re-accessing/multiple reconstructions likely both warp and reinforce memories, both real and false.

In conclusion, while it would be inaccurate to suggest that the core details of all memories are inherently unreliable, reconstruction and distortion of memory have been well established in the scientific literature. Although researchers may disagree about the rates of false-memory implantation across different methodological designs, the cumulative body of research, including Murphy, Dawson, et al.’s (^2023^) replication, provides compelling proof of principle that it *is possible* to implant false memories, which is the central issue.
